# Sodium humate alters the intestinal microbiome, short-chain fatty acids, eggshell ultrastructure, and egg performance of old laying hens

**DOI:** 10.3389/fvets.2022.986562

**Published:** 2022-10-12

**Authors:** Chenqinyao Li, Xue Li, Piwu Li, Bin Wei, Cong Zhang, Xiaoling Zhu, Jie Zhang

**Affiliations:** ^1^State Key Laboratory of Biobased Material and Green Papermaking, School of Bioengineering, Qilu University of Technology (Shandong Academy of Sciences), Jinan, China; ^2^Shandong Asia-Pacific Haihua Biotechnology Co., Ltd., Jinan, China; ^3^Shandong Academy of Agricultural Sciences, Jinan, China

**Keywords:** sodium humate, intestinal microbiome, SCFAs, trace element, older hens, egg quality

## Abstract

This study investigated the effect of sodium humate supplementation on changes in the intestinal microbiome, intestinal short-chain fatty acids production, and trace element absorption in older laying hens, with consequent effects on egg performance and shell quality. We used the same hens as their own control; a total of 720 laying hens aged 422 days were randomly divided into three replicates, with the CON group fed a commercial diet at 422–441 days of age and the HANa group fed a commercial diet supplemented with 0.05% sodium humate at 442–461 days of age. Compared with the CON group, in the HANa group, Bacteroidetes and Actinobacteria were significantly increased, whereas, Firmicutes was significantly decreased. Further, *Veillonella, Enterococcus, Lactobacillus*, and *Turricibacter* significantly decreased, and *Peptoniphilus, Helcococcus, GW-34, Psychrobacter, Anaerococcus, Corynebacterium, Facklamia, Trichococcus, Gallicola, Clostridium*, and *Oscillospira* were significantly increased. The results showed that sodium humate significantly altered the alpha and beta diversity and changed the structure of the intestinal microbiome. Acetic acid, isovaleric acid, and isobutyric acid, among short-chain fatty acids were significantly increased in the HANa group, whereas trace elements such as Mn, Zn, and Fe were significantly reduced. The eggshell strength and ultrastructure were significantly altered. In this study, sodium humate was found to alter the intestinal microbiome structure of aged hens, change the production of short-chain fatty acids, and promote the absorption of trace elements to keep aged hens from experiencing a decrease in egg production performance.

## Introduction

The intestinal microbiome of animals is closely associated with host nutrient metabolism and health (1, 2). Changes in the structure of the gut microbiome will directly affect the content of short-chain fatty acids (SCFAs), thereby affecting the intestinal absorption of nutrients (3). SCFAs are among the main metabolites produced *via* intestinal microbial fermentation and play important roles in suppressing the invasion of pathogenic microorganisms, promoting the development of intestinal villi, and enhancing nutrient absorption (4, 5).

Chickens and eggs provide essential nutrients for humans; however, egg production and quality are greatly influenced by hen age, the environment, and egg storage (6, 7). The establishment and maintenance of the intestinal microbiome are affected by multiple factors, and age is an important influencing consideration (8). As laying hens age, they tend to be characterized by a low egg production rate. Moreover, poor egg quality can be attributed to a decline in the intestinal microbiome, which limits nutrient absorption efficiency (9). There are notable differences with respect to the effects of different feeding strategies on the intestinal microbiome of laying hens (1, 10, 11). Dietary intervention has been shown to significantly regulate the structure and function of the intestinal microbiome community (12). Consequently, it is possible to enhance the nutritional digestion, absorption, metabolism, and overall health and growth performance of poultry by improving the structure of the gut microbiome (13–15).

Sodium humate is a macromolecular substance comprising humic acid, which is rich in phenolic hydroxyl and carboxyl groups and has antidiarrheal, antioxidant, and anti-inflammatory properties. Moreover, studies have shown that humic acid inhibits *Escherichia coli* and other pathogenic microorganisms (16–19). The effect of sodium humate (SH) on improving the gut microbiome and intestinal functions was demonstrated in experiments using weaned calves (20, 21), mice (22), tilapia (17), and piglets (23). However, it is unclear whether it would also have an effect on older laying hens.

In this study, we investigated the effects of supplementing the diets of aged hens with SH on the intestinal microbiome, SCFAs, trace element absorption, egg production performance, and egg quality. The primary objective was to provide a solution for the reduced egg production performance in older laying hens.

## Materials and methods

### Experimental design, diets, animal management, and sample collection

The Animal Care and Use Committee of the Qilu University of Technology approved all experimental procedures for the present study (#201907104).

In total, 720 healthy hens, 422 days of age, were born at the same time and kept in the same environment. We used the same hens as their own control for comparisons; the experiment was conducted for a total of 40 days based on a CON group (422–441 days of age) and HANa group (442–461 days of age). Hens were randomly divided into three replicates of 240 hens each. The CON hens were fed a basal diet (Table 1) based on maize and soybean meal, consistent with the Agricultural Trade Standardization of China (NY/T33–2004). The HANa group was fed a basal diet supplemented with SH (0.05%; Table 1). The SH used in this study was gifted by Shandong Asia-Pacific Haihua Biotechnology Co., Ltd., with a purity of 62.3%.

**Table 1:** Experimental diet composition.

**Ingredients, %**	**Experimental diets**	
	**CON**	**HANa**
Corn grain	63.17	63.17
Soybean meal	23	23
Wheat bran	2	2
Stone powder	6	6
Limestone	3.01	2.96
Soybean oil	1	1
70-Lysine	0.15	0.15
Calcium hydrogen phosphate	0.7	0.7
Layers multidimensional	0.03	0.03
0.2% Multi minerals for poultry	0.2	0.2
Sodium humate	0	0.05
Laying hens complex enzyme	0.03	0.03
NaCl	0.35	0.35
Methionine	0.15	0.15
50% Choline chloride	0.12	0.12
Betaine	0.05	0.05
10000 Phytase	0.03	0.03
VC	0.01	0.01
Total	100	100

All hens were housed in an environmentally controlled facility with the temperature maintained at approximately 24 ± 2 °C, in which ventilation and lighting (16L: 8D) were automatically controlled. All hens were supplied with feed and water *ad libitum*. At the end of each group of experiments, 20 laying hens were randomly selected from each replicate and were euthanized *via* cervical dislocation. The abdominal cavity was opened on a sterile table. The intestinal contents were collected into sterile tubes by gently squeezing the intestine (cecum) from the proximal to the distal end, treated with liquid nitrogen, and stored at −80°C as the sample. Each treatment group had three biological replicates. Each replicate experiment consisted of the intestinal contents of 20 hens.

### Intestinal microbiome

Total bacterial DNA was extracted from the intestinal content produced by hens in each group using a HiPure Stool DNA Kit (Magen, Guangzhou, China) and used as a template to amplify the V3-V4 variable regions of the 16S ribosomal RNA gene using specific primers (341F: 5′-CCTACGGGNGGCWGCAG-3′, 806R 5′-GGACTACHVGGGTATCTAAT-3′). Amplicons were extracted from 2% agarose gels and purified using an AxyPrep DNA Gel Extraction Kit (Axygen Biosciences, Union City, CA) according to the manufacturer's instructions, and then quantified using a StepOnePlus Real-Time PCR System (Applied Biosystems, Life Technologies, Thermo Fisher Scientific, Foster City, CA). Purified amplicons were pooled in equimolar concentrations and paired-end sequenced (PE250) using the Illumina platform (Personal Biotechnology, Shanghai, China) according to standard protocols. Raw sequence data were quality-screened and assembled using Flash software (24). UCHIME was used to identify interrogative sequences, eliminate chimeras, and count high-quality sequences (25). Clean tags were clustered into operational taxonomic units (OTUs) based on a 97% similarity level using Uparse (version 9.2.64). A Venn diagram was prepared to graphically represent common and unique OTUs among samples (26). Abundance and diversity indices were calculated and presented using R Qiime (27).

### SCFAs

SCFAs in the intestinal contents were determined using a Thermotrace 1310 Gas Chromatograph (Thermo Fisher Scientific, Waltham, MA). Briefly, 50 μL of 15% phosphoric acid, 100 μL of 125 μg/mL internal standard (isohexanoic acid) solution, and 400 μL of ether were added to 50 mg of sample, homogenized for 1 min, and centrifuged at 4 °C and 12,000 rpm for 10 min, after which the supernatant was removed for analysis. The chromatographic conditions were as follows: Agilent hp-innowax capillary column (30 m × 0.25 mm ID × 0.25 μm); (Agilent Technologies, Santa Clara, CA); split injection, injection volume of 1 μL, split ratio of 10:1. The injection port, ion source, transmission line, and quadrupole temperatures were 250, 230, 250, and 150 °C, respectively. The initial temperature of the programmed heating was 90 °C, which was then raised to 120 °C at a rate of 10 °C/min, followed by an increase to 150 °C at a rate of 5 °C/min, and finally to 250 °C at a rate of 25 °C/min for 2 min. The carrier gas was helium at a 1.0 mL/min flow rate. Mass spectrometry conditions were as follows: electron bombardment ionization source, single ion monitoring scanning mode, and electron energy of 70 eV.

### Trace elements

The trace elements in intestinal contents were determined using atomic absorption spectrometry (Thermo Fisher ice 3500; Thermo Fisher Scientific). Before determinations, fresh samples were dried at 65 °C, crushed, sieved through a 40 mesh, and stored in self-sealed bags. For analysis, a 1–2 g sample of dried feces was accurately weighed into a crucible and heated in an electric furnace until it was completely carbonized. Thereafter, the carbonized sample was placed in a muffle furnace and heated at 550 °C for 4 h until it was grayish-white in color. After cooling, 10 mL of concentrated nitric acid was added to the residual material, and the mixture was heated in an electric furnace until it was slightly boiling. After micro-boiling for 5 min, the sample was filtered into a 100 mL volumetric flask while still hot, and after cooling, it was made up to a volume.

### Egg quality and performance

Daily egg production, total egg weight, and feed consumption were recorded to calculate the daily egg production, egg production rate, and feed conversion rate. At the end of each experiment (CON and HANa), 100 eggs were randomly collected for egg quality analysis. After using distilled water to clean the surface of the eggshell using an Egg-Or-Candler (ORKA Food Technology, Bountiful, UT), photographs were captured, and observations were made. After that, the shell ratio (shell weight/egg weight) was measured using an EggAnalyze^®^ (ORKA Food Technology). An Eggshell Thickness Gauge (ORKA Food Technology) was used to ultrasonically measure the shell thickness, whereas eggshell strength was measured using an Egg Force Reader (ORKA Food Technology).

The internal and external surfaces of the shell were cleaned with distilled water. Pieces of eggshells approximately 0.5 cm^2^ in size were taken from the equatorial portion of the egg. Eggshell samples were fixed on a copper block, sprayed with gold powder, and viewed using a scanning electron microscope (SUPRA 55 Thermal Field Emission Scanning Electron Microscope; ZEISS, Oberkochen, Germany) for ultrastructural observations. Image pro plus (Media Cybernetics, USA) software was used to process images as data.

### Statistical analyses

Statistics were presented as the mean ± standard error of the mean. Statistical significance was evaluated by performing a two-tailed unpaired Student's *t*-test using GraphPad Prism 6.0. Statistical significance was set as ^*^ *p* ≤ 0.05, ^**^ *p* ≤ 0.01, and ^***^ *p* ≤ 0.001.

## Results

### Differences in intestinal microbiomes between groups at the phylum and genus level

**Figure 1:**
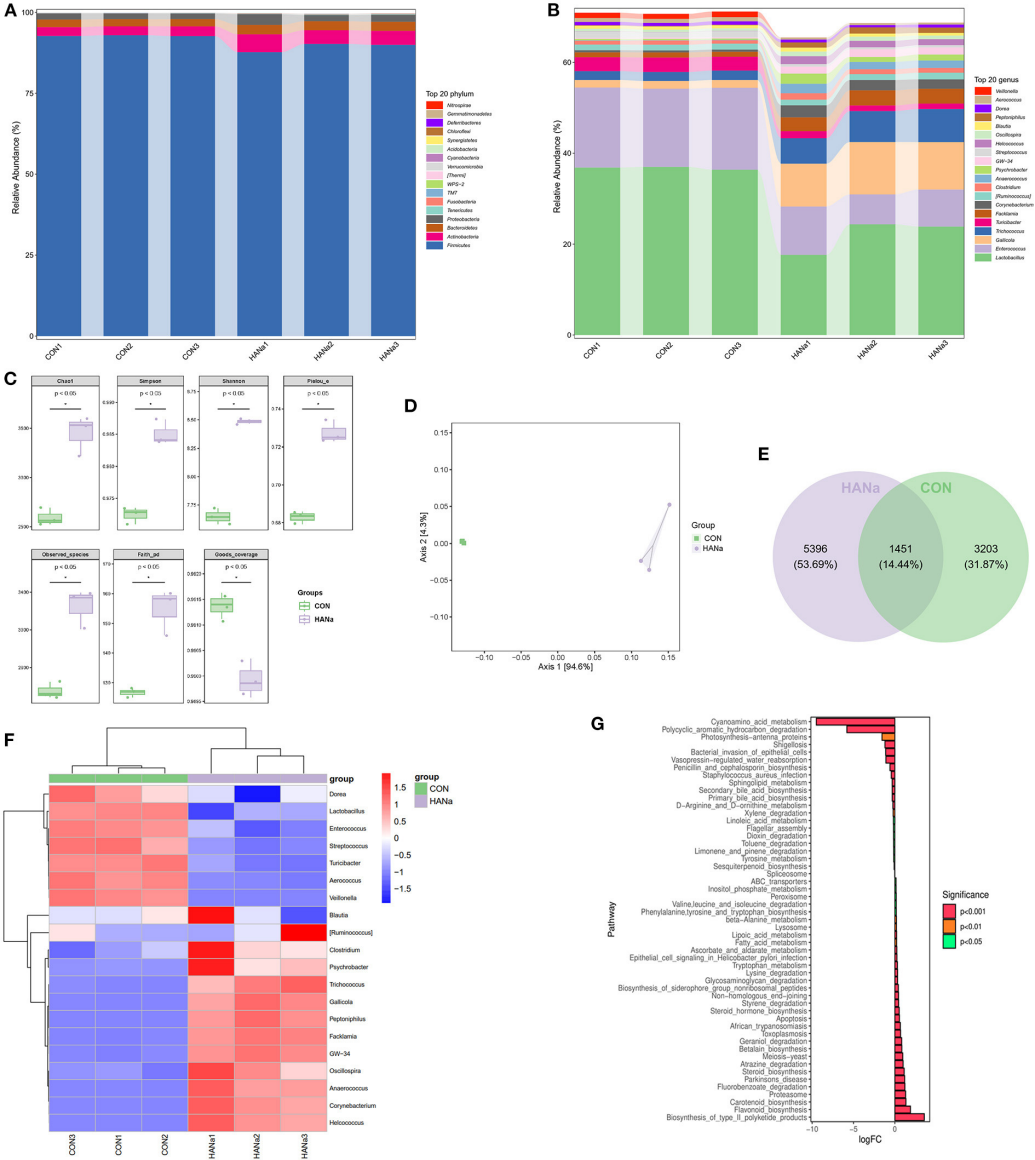
Effect of sodium humate on alterations in the intestinal microbiome structure of aged laying hens. **(A)** Composition of the top 20 phyla. **(B)** Composition of the top 20 genera. **(C)** Alpha diversity analysis of intestinal microbiomes. **(D)** Principal coordinates analysis (PCoA) of intestinal microbiome structures. **(E)** Venn diagram displaying the overlaps among groups. **(F)** Genus-level species composition heat map based on intestinal microbiome colony biclustering. **(G)** KEGG metabolic pathway analysis.

Compared with those in the CON group, in the HANa group, populations of Bacteroidetes and Actinobacteria showed a significant increase (*P* = 0.0005 and 0.01, respectively) whereas Firmicutes was significantly decreased (*P* = 0.013), and the abundance of Proteobacteria was increased (*P* = 0.207) (Figure 1A).

Among the top 20 genera in the gut microbial communities, *Veillonella, Enterococcus, Lactobacillus*, and *Turricibacter* (*P* = 0.000007, 0.0016, 0.0024, and 0.00014, respectively) were significantly decreased in the HANa group. In contrast, populations of *Peptoniphilus, Helcococcus, GW-34, Psychrobacter, Anaerococcus, Corynebacterium, Facklamia, Trichococcus, Gallicola, Clostridium*, and *Oscillospira* were increased (*P* = 0.000081, 0.000356, 0.000013, 0.023, 0.00039, 0.00034, 0.000017, 0.0008, 0.00013, 0.049, and 0.005, respectively) (Figure 1B).

### Alpha and beta diversity analysis

We next detected differences in Chao1, Simpson, Shannon, Pielou_e, Observed_species, Faith_pd, and Goods_coverage indexes between the HANa and CON groups (*p* < 0.05) (Figure 1C). SH contributed to microbial diversity in terms of alpha diversity. In addition, principal coordinate analysis (PCoA) of beta diversity showed that SH significantly changed the microbial community composition of the HANa group (Figure 1D).

### Species difference analysis

The Venn diagram shown in Figure 1E indicated that 3203 distinct OTUs were clustered in the CON group, with 5396 in the HANa group, indicating that SH increased the richness of the gut microbial community.

To further compare the species composition differences between the CON and HANa groups, a biclustering genus level species composition heat map was used for the analysis shown in Figure 1F, demonstrating that SH altered the intestinal microbiome of laying hens and changed the species and genera.

### Analysis of metabolic pathway differences

Phylogenetic investigation of communities by reconstruction of unobserved states analysis was then conducted to predict functional potential using software predict the functional abundance of samples based on the abundance of marker gene sequences in the samples (28). For the 16S rRNA data, we conducted a differential analysis based on KEGG metabolic pathway (Figure 1G). The function prediction results revealed that among pathways related to biosynthesis, type II polyketide products, flavonoids, carotenoids, steroids, betalains, steroid hormones, phenylalanine, tyrosine, and tryptophan were significantly up-regulated. Meanwhile, for the metabolic pathways, tryptophan, ascorbate, alternate, fatty acid, lipoic acid, beta-alanine, and inositol phosphate were significantly up-regulated. Furthermore among the pathways related to degradation, fluorobenzoate, atrazine, geraniol, styrene, glycosaminoglycan, valine, leucine, and isoleucine were significantly up-regulated. ABC transporters were also significantly up-regulated. The function prediction results revealed that among pathways related to biosynthesis, sesquiterpenoid, primary bile acid, secondary bile acid, penicillin, and cephalosporin were significantly down-regulated. For metabolic pathways, tyrosine, linoleic acid, D-Arginine and O-ornithine, sphingolipid, and cyanoamino acid, were significantly down-regulated. Limonene, pinene, toluene, dioxin, xylene, and hydrocarbon were significantly down-regulated among the degradation-related pathways. Moreover, *staphylococcus aureus* infection was significantly downregulated.

### Effects of SH on SCFAs in the intestinal content

**Figure 2:**
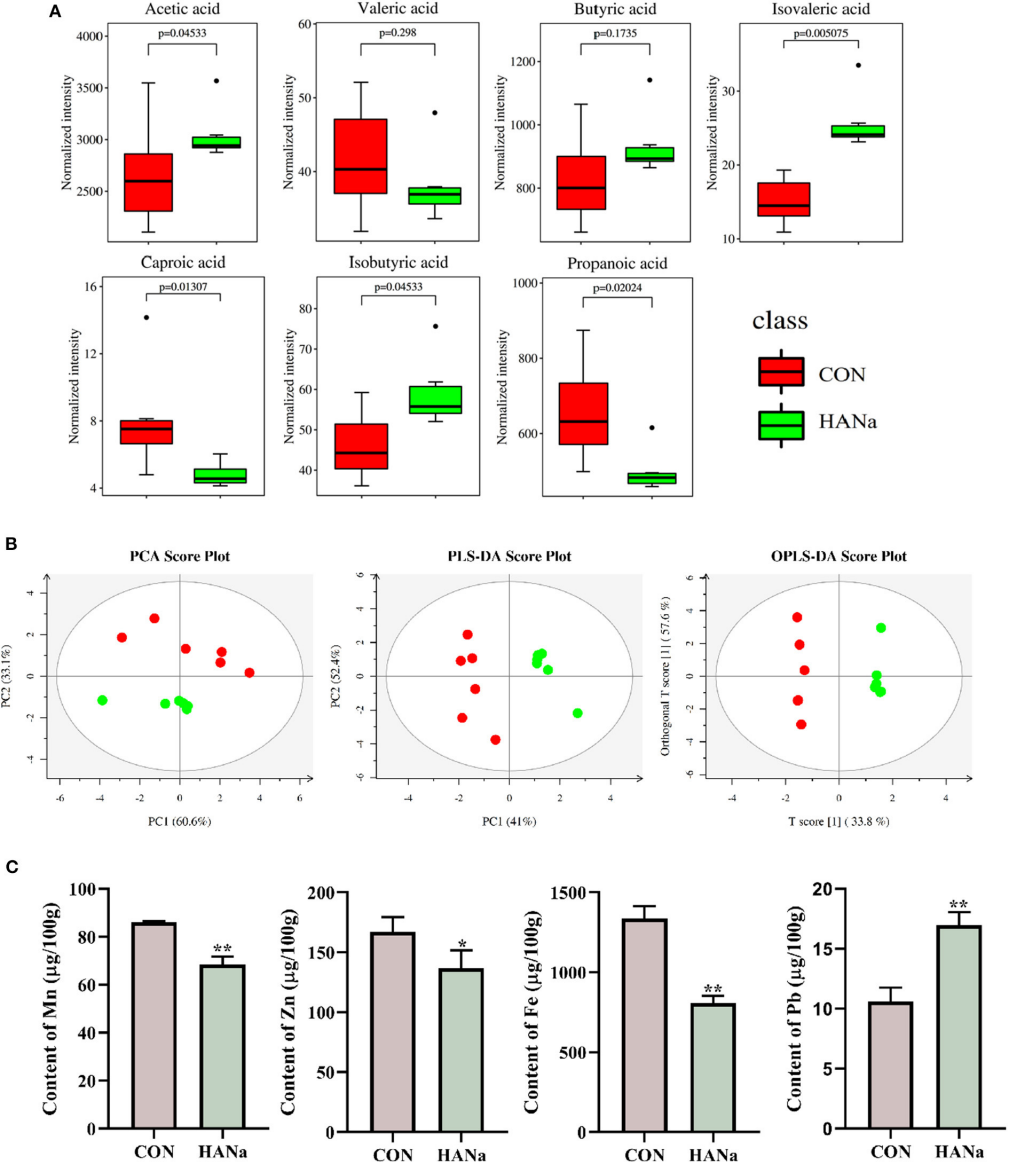
Effect of sodium humate on the content of short-chain fatty acids and trace elements in the intestinal contents of aged laying hens. **(A)** Short-chain fatty acid content. **(B)** Multivariate statistical analysis of short-chain fatty acids (principal component analysis, PCA; partial least squares-discriminant analysis, PLS-DA; orthogonal partial least squares discriminant analysis, OPLS-DA). **(C)** Contents of trace elements (Mn, Zn, Fe, and Pb). * *p* ≤ 0.05, ** *p* ≤ 0.01.

Compared with levels in the CON group, acetic acid, isobutyric acid, and isovaleric acid showed significant increases. In contrast, propionic acid, caproic acid, and valeric acid levels decreased in the HANa group, with propionic acid and caproic acid showing significant reductions (Figure 2A).

PCoA showed (Figure 2B) that the distribution within groups was relatively concentrated in the samples. In contrast, the distances and differences between the HANa and CON groups were significant, indicating that SH significantly changed the composition of SCFAs in the intestines of laying hens.

### Effects of SH on the contents of trace elements in the intestine

Compared to levels in the CON group, the HANa group showed a significant decrease in the trace elements Mn, Zn, and Fe in the intestinal content of the old laying hens and a significant increase in Pb (Figure 2C).

### Productive performance and eggshell quality

**Figure 3:**
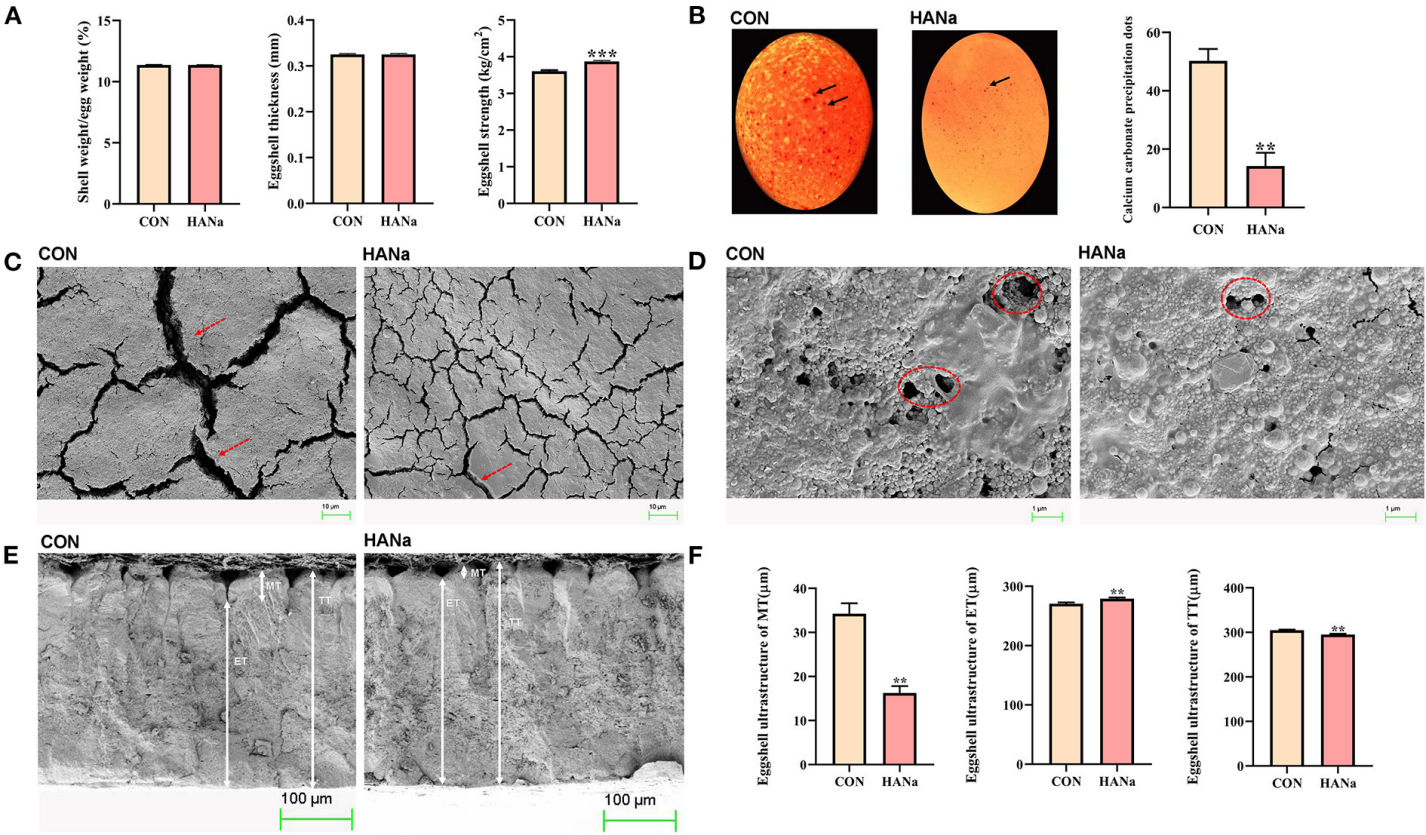
Effect of sodium humate on eggshells. **(A)** Egg and eggshell quality in laying hens. **(B)** Precipitation of calcium carbonate on the surfaces of eggshells. **(C)** Observation of the ultrastructure of the eggshell surfaces in 1.00 KX. **(D)** Observation of the ultrastructure of eggshell surfaces in 10.00 KX. **(E)** Observation of the ultrastructure of eggshell cross-sections. **(F)** Effects of sodium humate on eggshell ultrastructure. Solid black arrows, calcium carbonate precipitation on the eggshell surface; red dashed arrows, cracks on the eggshell surface; red dashed circles, stomata on the eggshell surface. MT, mammillary layer thickness; ET, effective layer thickness; TT, total thickness. ** *p* ≤ 0.01 and *** *p* ≤ 0.001.

Dietary supplementation with SH did not significantly affect egg production, the egg production rate, and the feed egg ratio (Table 2). Values obtained for the related indices of egg quality, namely the eggshell ratio (shell weight/egg weight), eggshell thickness, and eggshell strength, are presented in Figure 3A. Dietary SH had a significant effect on shell strength. The results showed that calcium carbonate precipitates were found on the eggshell surface in the CON group, and a significant decrease in calcium carbonate precipitates was observed in the HANa group (Figure 3B).

**Table 2:** Effects of sodium humate on productive performance.

**Variable**	**CON**	**HANa**	***P*** **-value**
Egg production (g/hen, day)	55.00 ± 0.060	55.49 ± 0.219	0.098
Egg production rate (%, each egg/hen, day)	86.21 ± 0.099	86.97 ± 0.343	0.099
Feed egg ratio	2.07 ± 0.007	2.05 ± 0.009	0.145

Eggshell ultrastructure was observed using electron microscopy. We observed the morphology of cracks and stomata on the eggshell surface at the same magnification. The results showed that the eggshell surface cracks were significantly narrower, and the stomata showed a more regular round shape in the HANa group compared with observations in the CON group at the same magnification (Figures 3C,D). Ultrastructural observations of the cross-meeting of the eggshell revealed that the mammillary layer thickness in the HANa group was significantly reduced, the effective layer thickness was significantly increased, and the total thickness was significantly reduced (Figures 3E,F).

## Discussion

The laying performance of hens is a commercially important aspect of reproductive performance. In this regard, the laying rate, eggshell quality, and egg nutrient contents of laying hens decrease with age after the peak laying period (29, 30). In this study, we found that SH supplementation in the diets of laying hens significantly altered the intestinal microbiome structure and the content of SCFAs in older laying hens. It was also found that SH promoted the absorption of trace elements in laying hens while adsorbing heavy metals that could not be avoided within the diets. SH also affected the weakened egg production performance observed with increasing age, and eggshells became stronger, as evidenced by their morphology and ultrastructure.

The intestinal tract of poultry has a very complex microbial community (31). We found that dietary supplementation with SH could help improve the gut microbiomes in older hens. In literature reports, Bacteroidetes, Firmicutes, Proteobacteria, and Fusobacteria form most of the microbiota during the peak period (32–34). In addition, the relative abundance of Bacteroidetes overtakes Firmicutes and becomes the dominant phylum in the late laying period (33). Compared with levels in the CON group, in the HANa group, although Bacteroidetes was significantly increased and Firmicutes was significantly decreased, Firmicutes remained the predominant phylum. Alpha and Beta diversity analyses showed higher gut microbial abundance and significant changes in microbial composition. A high alpha-diversity has been associated with superior host health, and a heightened diversity is one of the factors contributing to good resistance to invasion by foreign pathogens (35).

In the HANa group of hens, a significant increase was observed in the abundance of *Oscillospira*, a genus involved in the decomposition of complex carbohydrates; this influences the metabolism of nutritional fiber, which is closely related to host health (36, 37). *Turicibacter* is believed to play important roles in intestinal mucus degradation and immune functions (38), and reductions in the abundance of *Turicibacter* have been observed in the intestines of mice undergoing voluntary exercise, concomitant with a reduction in colitis (39). In the HANa group hens examined in the present study, we observed a reduction in the abundance of *Turicibacter* (*P* = 0.00014). Although bacteria in the genus *Enterococcus* were initially identified as harmonious symbionts in the gastrointestinal tracts of chicks, over the past 15 years, pathogenic *Enterococcus decorum* has become an important cause of morbidity and mortality in broiler breeders (40). We detected a reduction in the abundance of *Enterococcus* in the HANa hens (*P* = 0.0016). Significant changes in the structure of the intestinal microbiome of old laying hens were observed in the HANa group. Accordingly, the KEGG results showed that *staphylococcus aureus* infection was significantly downregulated.

It has been shown that numerous intestinal bacteria can synthesize SCFAs using dietary fiber as a substrate (41). SCFAs are essential for intestinal health and nutrient absorption. Hence it is desirable to enhance the abundance of SCFA-producing microorganisms and thereby promote the synthesis of SCFAs in the intestines. Compared with that in the CON group, the abundance of Bacteroidetes and Actinobacteria in the HANa group hens increased significantly. Bacteroidetes can convert monosaccharides produced by the decomposition of complex carbohydrates to SCFAs, thus promoting host metabolism, such as regulating lipid metabolism and altering endocrine function (2, 42). Furthermore, Bacteroidetes species are the primary producers of acetate and propionic acid (43). Actinobacteria is another important phylum comprising SCFA-producing bacteria, including those that can degrade dietary carbohydrates (44, 45). At the genus level, we detected significant increases in the abundances of *Clostridium* and *Psychrobacter* (*P* = 0.049 and 0.023, respectively). Studies have shown that the main metabolites of *Clostridium* are butyrate, propionic acid, and acetic acid (45, 46). Among enzyme activities characteristic of *Psychrobacter* is carbonic anhydrase, which has potential applications for bioremediation (47, 48). Carbonic anhydrase activity is associated with the formation and secretion of carbonate during eggshell development. Thus, an increase in microbially synthesized carbonic anhydrase can further improve eggshell quality (49, 50). *Oscillospira* bacteria can produce butyrate and prevent *Clostridium difficile* infection, which is similar to the effects of other genera, such as *Roseburia* and *Faecalibacter* (51, 52). In the HANa group hens, we detected a significant increase in the abundance of carbohydrate-degrading bacteria of the genus *Trichococcus*, which contain numerous enzymes associated with the conversion of pyruvate to ethanol, acetate, and lactate (53). An analysis of KEGG metabolic pathways revealed that the degradation pathways of certain amino acids (valine, leucine, and isoleucine) were significantly up-regulated. The findings of previous studies have consistently indicated that the consumption of amino acids leads to an increase in SCFAs (54).

Studies have shown that intestinal microbial metabolites play essential roles in the human body. For example, regulating the content and types of SCFAs can prevent various diseases (55, 56). SCFAs are the primary energy source of intestinal epithelial cells, and they can promote the proliferation of intestinal epithelial-absorbing cells and play an important role in the intestinal energy supply. They also maintain intestinal mucosal barrier functions, regulate intestinal hypersensitivity and immunity, and exert anti-tumor effects (57). Acetic acid, propionic acid, and butyric acid are the main SCFAs and occur at a ratio of 3:1:1 (58), among which acetic acid can inhibit gastric cell apoptosis and promote mucin production (59). In the present study, we detected an increase in the acetic acid content in HANa group hen feces (*P* = 0.045). Butyric acid is one of the most critical intestinal energy sources and plays a vital role in promoting intestinal development and maintaining the integrity of intestinal epithelial cells (42). Butyrate also promotes the production of mucin, which leads to changes in bacterial adhesion ability (60). In this regard, studies have shown that bacteria of the genus *Clostridium* mainly produce butyric acid (46), propionic acid, and acetic acid (45), whereas those of the phylum Bacteroidetes primarily produce acetic acid and propionic acid (43). In the present study, we observed a significant reduction in intestinal propionic acid content compared with increases in acetic acid and butyric acid. Propionic acid is primarily produced *via* two pathways, the succinic acid pathway in Bacteroidetes and the lactic acid pathway in Firmicutes (61). There are relatively few intestinal microorganisms that can synthesize propionic acid, among which are species of *Veillonella*, which can convert lactic acid to propionic acid (62). We found that the abundance of *Veillonella* in the HANa group hens was significantly lower than that in the hens of the CON group, which might be the reason for the decrease in propionic acid. An increase in acetic acid and butyric acid provides energy for intestinal villi and promotes villus cell proliferation and development. This not only improves the barrier function of the intestinal mucosa but also reduces the invasion of pathogenic microorganisms and harmful chemicals and enhances the nutrient absorption efficiency of the intestinal wall.

The analysis of trace elements in hen feces indicated that compared with levels in the CON group, there was a significant increase in Mn, Zn, and Fe utilization in the HANa group hens. Mn and Zn contribute to enhancing eggshell quality (63), and Fe contributes to enhancing the transport capacity of nutrients and oxygen within the blood, promoting the development of the reproductive system and further enhancing its function in the process of eggshell assembly (64). Although the contents of heavy metals in raw feed materials and additives are limited, animals will inevitably ingest specific amounts of heavy metals from feed products (65). However, residual heavy metal elements in the body and animal products can be minimized by adopting specific measures to promote fecal excretion of the heavy metal contents from basal diets. In the present study, we found that compared with that in hens of the CON group, the content of Pb in the HANa group hens increased from 0.106 mg/kg to 0.169 mg/kg. This 59.43% increase can be attributed primarily to the fact that humic acid adsorbs Pb in feed, which is subsequently excreted in the feces, thereby reducing the deposition of Pb in the body and eggs of laying hens. Indeed, given its absorptive properties, SH is often used to remove heavy metals consumed in dietary sources (66).

The factors contributing to the eggshell defects observed in older laying hens are complex. However, it is conceivable that with increasing age, the eggs laid by hens become more extensive and the shells become thinner (67). We found that calcium carbonate precipitation on the surface of eggshells supplemented with SH was significantly reduced based on observations of egg morphology. The strength of the eggshells also increased significantly. The eggshell ultrastructure was also significantly altered in the HANa group. The ultrastructures of eggshell cross-sections revealed a greater effective thickness and a decrease in the mastoid thickness, which contributes to a stronger ultrastructure (68). The absorption of Mn and Zn helps to increase papillary node density in the early deposition phase of eggshell formation, contributing to a more robust eggshell structure (50, 69). The cracks and pores on the eggshell surface also indicate a strengthened eggshell structure in the HANa group.

## Conclusion

**Figure 4:**
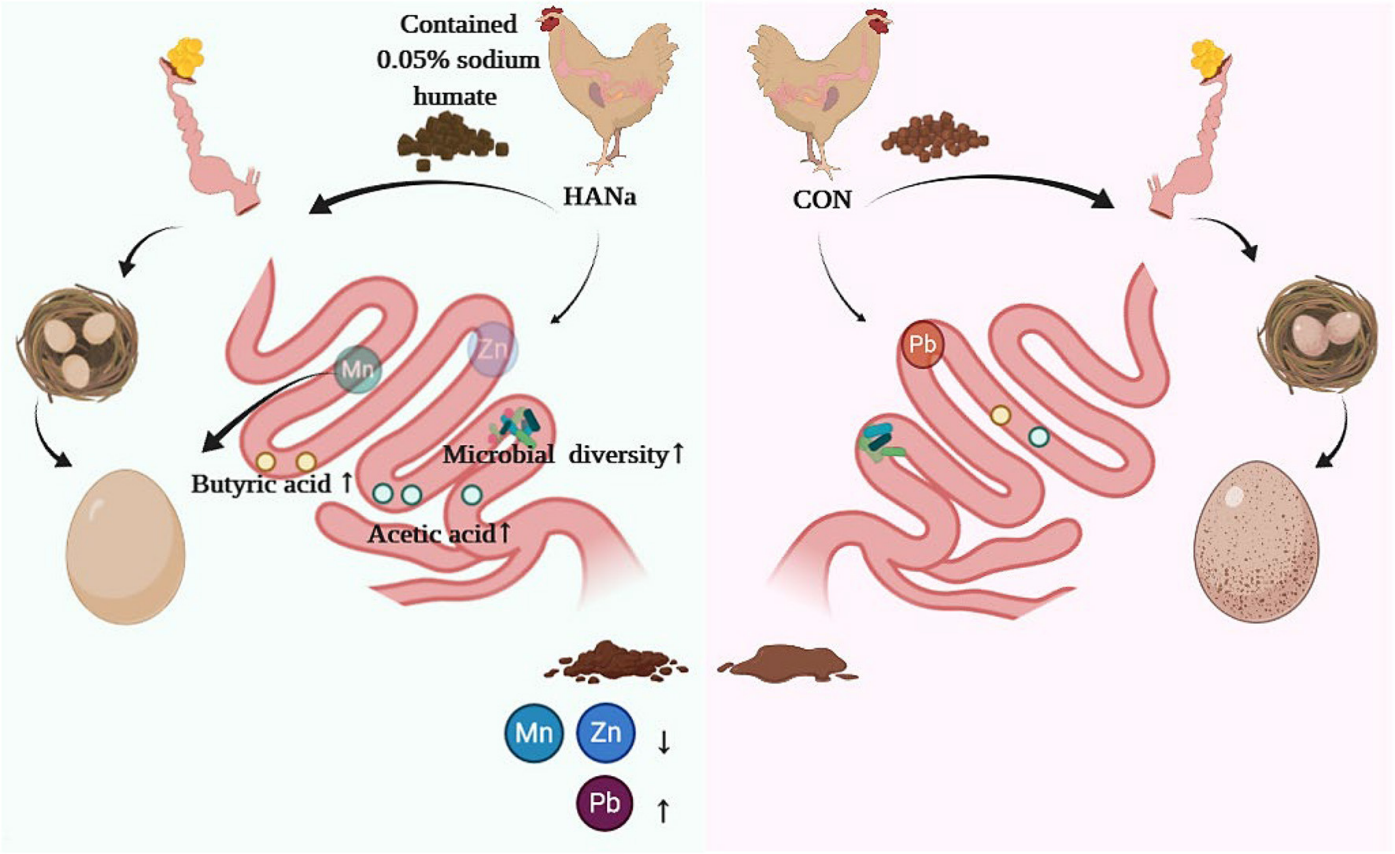
Schematic diagram showing how sodium humate alters the intestinal microbiome of old hens to improve the formation of short-chain fatty acids and the utilization of trace elements.

In summary, SH supplementation as a feed additive in the late laying period can increase SCFAs contents and promote the absorption of trace elements (Mn, Zn, and Fe) *via* its effects on altering the abundance of the intestinal microbiome, thereby maintaining the production performance of laying hens and enhancing eggshell quality (Figure 4).

## Data availability statement

The datasets presented in this study can be found in online repositories. The names of the repository/repositories and accession number(s) can be found below: https://www.ncbi.nlm.nih.gov/, PRJNA672821.

## Ethics statement

The animal study was reviewed and approved by the Animal Care and Use Committee of the Qilu University of Technology.

## Author contributions

JZ and CL were involved in the experimental design and drafted and edited the manuscript. PL and XZ reviewed the literature and conducted statistical analyses. BW and CZ collected the data. CL and XL conducted chemical analyses. All authors contributed to the article and approved the submitted version.

## Funding

The work was supported by the Natural Science Foundation of Shandong Province (Grant number ZR2020KE038), Qilu University of Technology of Cultivating Subject for Biology and Biochemistry (Grant number 202019), and Shandong Province Agricultural Major Application Technology Innovation Project (Grant number 20182130106).

## Conflict of interest

Authors BW and CZ were employed by Shandong Asia-Pacific Haihua Biotechnology Co., Ltd. The remaining authors declare that the research was conducted in the absence of any commercial or financial relationships that could be construed as a potential conflict of interest.

## Publisher's note

All claims expressed in this article are solely those of the authors and do not necessarily represent those of their affiliated organizations, or those of the publisher, the editors and the reviewers. Any product that may be evaluated in this article, or claim that may be made by its manufacturer, is not guaranteed or endorsed by the publisher.
